# ELDER SEXUAL ABUSE: A REVIEW OF LEGALLY MANDATED APPROACHES TOWARD PREVENTION AND FUTURE IMPLICATIONS

**DOI:** 10.1093/geroni/igz038.3510

**Published:** 2019-11-08

**Authors:** Michelle Hand

**Affiliations:** The Ohio State University, Columbus, Ohio, United States

## Abstract

Rape stereotypes involve the assault of young women due to sexual desire, resulting in the exclusion of older adults from sexual violence research, policies, and interventions, suggesting a need to further knowledge in this area (Bows & Westmarland, 2015). Debates also persist on the prevalence and nature of elder sexual abuse (ESA), where it occurs, and its most common perpetrators (Bows, 2018). Thus a systematic scoping review was conducted to explore the nature and prevalence of ESA as well as mandated prevention and intervention strategies to guide practice, policy, research and education on prevention. Eligible sources were research-based and focused on the nature of ESA along with legal prevention and/or intervention mandates. In total, 38 peer-reviewed articles and reports were screened in for review, obtained from AgeLine, EBSCO, Clinical Key Flex, PubMed, Google, and Google Scholar databases. Findings suggest while consensus has not been reached (Bows, 2018), ESA estimates range from 0.2% to 7% of U.S. elder abuse cases, yet actual rates are likely much higher due to underreporting (Cannell et al., 2014). Additionally, results suggest ESA most often occurs in nursing homes, predominantly perpetrated by staff or residents (Ramsey-Klawsnik et al., 2008). Still, ESA remains underreported despite several mandated approaches to prevention and intervention (Payne, 2010). Thus findings demonstrate a need for reliable estimates of prevalence, location as well as common victim-perpetrator relations and awareness of required steps toward prevention and intervention (Payne, 2010). Beyond this, transdisciplinary efforts are needed to yield effective training, education, and culturally appropriate resources.

